# The locations of stroke lesions next to the posterior internal capsule may predict the recovery of the related proprioceptive deficits

**DOI:** 10.3389/fnins.2023.1248975

**Published:** 2023-10-03

**Authors:** Thomas Hassa, Monika Zbytniewska-Mégret, Christian Salzmann, Olivier Lambercy, Roger Gassert, Joachim Liepert, Mircea Ariel Schoenfeld

**Affiliations:** ^1^Lurija Institute for Rehabilitation and Health Sciences, University of Konstanz, Konstanz, Germany; ^2^Neurological Rehabilitation Center Kliniken Schmieder, Allensbach, Germany; ^3^Rehabilitation Engineering Laboratory, Department of Health Sciences and Technology, Institute of Robotics and Intelligent Systems, ETH Zurich, Zurich, Switzerland; ^4^Future Health Technologies, Singapore-ETH Centre, Campus for Research Excellence and Technological Enterprise (CREATE), Singapore, Singapore; ^5^Department of Neurology, Otto-von-Guericke-University Magdeburg, Magdeburg, Germany; ^6^Department of Behavioral Neurology, Leibniz-Institute for Neurobiology, Magdeburg, Germany; ^7^Neurological Rehabilitation Center Kliniken Schmieder, Heidelberg, Germany

**Keywords:** stroke rehabilitation, sensorimotor performance, voxel-based lesion-symptom mapping (VLSM), somatosensory evoked potentials (SSEP), robotic assessment, proprioception, predictor, recovery

## Abstract

**Background:**

Somatosensory deficits after stroke correlate with functional disabilities and impact everyday-life. In particular, the interaction of proprioception and motor dysfunctions affects the recovery. While corticospinal tract (CST) damage is linked to poor motor outcome, much less is known on proprioceptive recovery. Identifying a predictor for such a recovery could help to gain insights in the complex functional recovery processes thereby reshaping rehabilitation strategies.

**Methods:**

50 patients with subacute stroke were tested before and after neurological rehabilitation. Proprioceptive and motor impairments were quantified with three clinical assessments and four hand movement and proprioception measures using a robotic device. Somatosensory evoked potentials (SSEP) to median nerve stimulation and structural imaging data (MRI) were also collected. Voxel-based lesion-symptom mapping (VLSM) along with a region of interest (ROI) analysis were performed for the corticospinal tract (CST) and for cortical areas.

**Results:**

Before rehabilitation, the VLSM revealed lesion correlates for all clinical and three robotic measures. The identified voxels were located in the white matter within or near the CST. These regions associated with proprioception were located posterior compared to those associated with motor performance. After rehabilitation the patients showed an improvement of all clinical and three robotic assessments. Improvement in the box and block test was associated with an area in anterior CST. Poor recovery of proprioception was correlated with a high lesion load in fibers towards primary sensorymotor cortex (S1 and M1 tract). Patients with loss of SSEP showed higher lesion loads in these tracts and somewhat poorer recovery of proprioception. The VSLM analysis for SSEP loss revealed a region within and dorsal of internal capsule next to the posterior part of CST, the posterior part of insula and the rolandic operculum.

**Conclusion:**

Lesions dorsal to internal capsule next to the posterior CST were associated with proprioceptive deficits and may have predictive value. Higher lesion load was correlated with poorer restoration of proprioceptive function. Furthermore, patients with SSEP loss trended towards poor recovery of proprioception, the corresponding lesions were also located in the same location. These findings suggest that structural imaging of the internal capsule and CST could serve as a recovery predictor of proprioceptive function.

## 1. Introduction

Motor as well as somatosensory capacities are mandatory for proper functioning of the hand and arm in daily activities. Seemingly pure motor tasks such as gripping or reaching movements require afferent somatosensory proprioceptive feedback. Motor learning is also heavily affected by proprioceptive deficits (Yousif et al., 2015; Miall et al., 2018). Somatosensory impairment is common after stroke and was reported in up to 85% of patients with unilateral stroke (Kim and Choi-Kwon, 1996). In a recent study (Kessner et al., 2019) 60% of patients with unilateral stroke showed symptoms in at least one somatosensory modality (light touch 39%, proprioception 28%). In another cohort, up to 64% of all individuals with stroke showed impaired proprioception (Connell et al., 2008).

The degree of weakness and stroke severity are significantly associated with sensory impairments (Tyson et al., 2008), and recovery from somatosensory deficits were reported to be of major importance for everyday living skills after stroke (Meyer et al., 2014). While the sensory deficit itself impacts daily life, proprioception deficits may also impair motor functions and reduce functional abilities and independence (Rand, 2018). Therefore, proprioceptive training is an important part of rehabilitation interventions after stroke (Doyle et al., 2010; Pollock et al., 2014).

Different lesion locations such as thalamus, insula, internal capsule, pons and cortical somatosensory areas were reported to be associated with somatosensory impairments (Kim, 1992; Baier et al., 2014). Whereas lesions in pons, internal capsule and partly the thalamus directly interrupt somatosensory input, defects in cortical areas such as insula and the secondary somatosensory cortex S2 affect the processing of this input.

For motor recovery it is well established that lesions in the corticospinal tract predict poor motor outcome (Lin et al., 2019; Liu et al., 2020). However, much less is known on their impact on somatosensory recovery. To date, there is only weak evidence on specific lesions interfering with the restoration of somatosensory performance, and which lesions may serve as a predictor for good or bad recovery. Some studies explored the impact of lesion location on the degree of somatosensory impairment (Kenzie et al., 2016; Meyer et al., 2016; Findlater et al., 2019; Kessner et al., 2019; Ingemanson et al., 2019a). Yet, only one study (Findlater et al., 2019) found a moderate correlation of proprioceptive scores with the lesion load in the corticospinal tract. To the best of our knowledge, this is the only reference describing a lesion location predictive for proprioceptive recovery.

Motor and proprioceptive deficits mostly occur together after stroke and there is a significant correlation between proprioceptive deficits and motor dysfunction (Yu et al., 2021). However, the relationship between motor and proprioceptive recovery is not fully understood yet. There are reports that severe somatosensory impairment in subacute stroke does not directly compromise motor recovery. Nevertheless, spontaneous somatosensory recovery seems to be a precondition for regaining full motor abilities (Zandvliet et al., 2020). There seem to be different timelines for proprioceptive and motor recovery, thus suggesting no or little direct effect of proprioceptive recovery on motor outcome (Semrau et al., 2015). Nevertheless, there is also evidence that the extent of proprioceptive deficits is related to the outcome for motor recovery after stroke (Meyer et al., 2014; Yoon et al., 2021). A deeper understanding of the processes involved in the recovery of motor and proprioceptive functions would help to improve rehabilitative interventions. Identifying a reliable predictor for proprioceptive recovery could be an important piece of the puzzle and further shape rehabilitation strategies.

The aim of the present study was to investigate the relationship between lesion location/lesion load and the associated motor and proprioceptive impairments of the upper extremity in subacute stroke patients. To characterise the impairments, we used clinical tests but also precise robot-based assessments, given that robotic measures of proprioception deficits were reported to be more sensitive than standard scales (Ingemanson et al., 2019a). Voxel-based lesion-symptom mapping was used to assign lesion areas to the specific impairment. Region of interest (ROI)-analysis was employed to test for regions previously described in other studies as being related to motor and proprioceptive deficits. We used lesion metric analysis (Findlater et al., 2019) on a subset of tracts (Archer et al., 2018) within the CST to improve spatial resolution. The pre-existing CST template used for the analysis (see Methods) was created using diffusion tensor imaging (DTI) and includes not only descending but also ascending fibers due to methodological limitations of DTI. This is evidenced by the fact that the S1 tract, which represents the fiber connections to the primary sensory cortex, lies entirely within this CST template. The CST template thus also includes ascending fibers, at least in the posterior part. The term CST is used in the following with reference to this CTS template and expressly not to the neuroanatomical definition of CST with exclusively descending fibers.

Additionally, we analysed structural correlates associated with loss of somatosensory evoked potentials (SSEP) related to poor functional outcome (Tzvetanov and Rousseff, 2003; Tzvetanov et al., 2005; Hwang et al., 2016).

## 2. Methods

### 2.1. Participants

50 participants with a subacute stroke were recruited consecutively as in-patients during their neurorehabilitation. The inclusion criteria were age > 18 years, less than 3 months post-stroke (ischemic or haemorrhagic) and the absence of contractures of the affected hand (passive flexion of the metacarpophalangeal joint (MCP) of the index finger by at least 20°). Exclusion criteria were: pain during movement of the MCP joint, presence of visuospatial neglect (Bells Test; Gauthier et al., 1989) or aphasia. All participants gave written informed consent before participating in the study. The study was approved by the Ethics Commission of Baden-Württemberg (F-2016-126) and registered as a clinical trial.

For 45 of these 50 patients (16 women, 29 men, with a mean age of 68.6 years ±10.5; range 40.5–86.6 years) we could obtain the structural imaging data (44 MRIs, 1 CCT). The mean interval between the stroke and imaging examination was 11.4 days ± 18.0; range 0–70 days. In 23 patients the lesion was right-sided, in 22 patients left-sided.

### 2.2. Clinical, robotic and neurophysiological measurements

Measurements were collected at study inclusion (baseline, T1) and after 4 weeks (discharge, T2), unless discharge from the clinic occurred earlier (5 patients), in which case the measurement was performed at the time of discharge (at least 2 weeks after inclusion). Only data from the affected side were considered for analysis.

The following clinical assessments were performed: Fugl-Meyer Upper Limb Motor Assessment (FMA; Gladstone et al., 2002) for the assessment of motor function (scores: 0–66), kinaesthetic Up-Down test as a part of the Nottingham Sensory Assessment (kUDT; Lincoln et al., 1998) for the assessment of finger proprioception (score: 0–3), Box and Block Test (BBT; Mathiowetz et al., 1985) for the assessment of functional hand use (unit: blocks/min).

For the robotic assessments, the patients were seated in front of the ETH MIKE (Motor Impairment and Kinaesthetic Evaluation) apparatus (Zbytniewska et al., 2021), grasping a handle with their index finger stretched and attached to the robot end-effector with straps. The robot could, depending on the assessment task, provide accurate one degree-of-freedom displacement of the index finger at the MCP joint and measure the displacement and interaction force as well as participant’s responses provided via a tablet. The direct view on the finger was blocked by a visual display of the tablet.

In this study, four different, previously validated (Zbytniewska et al., 2021) assessment tasks were performed. One task assessed proprioception: (a) Position Matching (PM); the outcome measure is the error in ° between actual and indicated positions after the finger was passively moved by the robot, and therefore does not require active movement of the tested finger (11 trials, randomized positions within a range of 10–30° from starting position). Three other tasks gauge distinct aspects of motor function: (b) Maximum Fingertip Force in Flexion: the participants pressed as strong as possible with the index finger in flexion direction (Force in Newton, mean value of 3 trials); (c) Active Range of Motion (ARoM): maximum degree of flexion and extension of index finger in ° (3 trials); (d) Maximum Velocity in Extension (Velocity): after being moved passively to a starting position in flexion, the participants extended the index finger as fast as possible to a target position displayed on the visual screen; the outcome measure was the mean of three maximum velocity values across the 5 trials performed (°/s from starting position in flexion). For additional details on the robotic assessments tasks and their outcome measures, please refer to (Zbytniewska et al., 2021).

A standard neurophysiological protocol was used to obtain SSEP by electrically stimulating the median nerve at the level of the wrist, and scalp recordings were taken from C3’ and C4’.

### 2.3. Analysis of behavioral data

Statistical analyses were conducted using IBM SPSS Statistics 29. Behavioral data and the results of the lesion mapping (voxel count; see below) were analyzed non-parametrically, when the Shapiro–Wilk Tests indicated that the data were not normally distributed. For paired samples the Wilcoxon test was used, and for two samples the Mann–Whitney U test (2 sample *t*-test and paired *t*-test for normally distributed data). Measures from clinical and robotic tests between timepoint T1 and T2 (Delta^T2-T1^) indicating proprioceptive and motor function changes were calculated by subtracting the absolute values at T1 from those at T2 (Tables 1–3).

**Table 1:** Behavioral data: clinical and robotic assessments at timepoint T1 (baseline) and T2; paired-T-Test/Wilcoxon between T1 and T2 for these assessments.

	Baseline T1		Discharge T2		Paired *T*-Test/Wilcoxon	
	Mean	SD	Mean	SD	*p*-value	Z-/*T*-value
FMA [0–66]	31.96	±23.10	38.37	±22.99	<0.001^**^	Z − 4.995
kUDT [0–3]	2.02	±1.16	2.24	±1.02	0.008^**^	Z − 2.640
BBT (#/min)	18.38	±19.93	26.24	±23.35	<0.001^**^	Z − 4.257
PM (°)	13.18	±5.51	11.82	±6.36	0.026^*^	T 2.003
ARoM (°)	51.86	±24.19	57.2	±24.81	0.005^**^	Z − 2.782
Force (N)	12.92	±12.68	15.47	±12.48	0.002^**^	Z − 3.082
Velocity (°/sec)	170.76	±176.77	192.54	±178.94	0.171	Z − 1.369

FMA: Fugl-Meyer Upper Limb Motor assessment; kUDT: kinaesthetic Up-Down test; BBT: Box and Block test; PM: position matching; ARoM: active range of motion; N: Newton.

* Significant correlation *p* < 0.05.

** Significant correlation *p* < 0.01.

**Table 2:** Corticospinal lesion metrics: correlations of two different lesion metrics (weighted lesion load and absolute overlap of stroke lesion maps with tract templates) with behavioral assessments at timepoint T1 and Delta^T2-T1^ (difference between values of timepoint T1 and T2).

			Absolute overlap					Weighted lesion load				
			S1	M1	SMA	preSMA	PMd	S1	M1	SMA	preSMA	PMd
FMA	T1	p	0.003	0.001	0.001	0.029	0.003	0.004	0.000	0.001	0.028	0.001
		Rho	−0.452^**^	−0.499^**^	−0.490^**^	−0.337*	−0.449^**^	−0.434^**^	−0.541^**^	−0.486^**^	−0.339*	−0.483^**^
	Delta^T2-T1^	p	0.281	0.288	0.667	0.910	0.638	0.630	0.657	0.579	0.763	0.793
		Rho	0.177	0.175	0.071	0.019	0.078	0.080	0.074	0.092	0.050	0.043
kUDT	T1	p	0.000	0.000	0.007	0.147	0.018	0.000	0.000	0.004	0.093	0.017
		Rho	−0.688^**^	−0.705^**^	−0.411^**^	−0.228	−0.364^*^	−0.657^**^	−0.584^**^	−0.439^**^	−0.263	−0.366^*^
	Delta^T2-T1^	p	0.172	0.062	0.736	0.693	0.979	0.103	0.068	0.504	0.931	0.954
		Rho	0.223	0.302	0.056	−0.065	0.004	0.265	0.296	0.110	−0.014	0.009
BBT	T1	p	0.000	0.000	0.000	0.048	0.003	0.000	0.000	0.000	0.042	0.001
		Rho	−0.527^*^*	−0.596^**^	−0.525^**^	−0.307^*^	−0.452^**^	−0.524^**^	−0.629^**^	−0.533^**^	−0.315^*^	−0.488^**^
	Delta^T2-T1^	p	0.408	0.167	0.077	0.220	0.291	0.270	0.083	0.064	0.213	0.128
		Rho	−0.136	−0.226	−0.286	−0.201	−0.173	−0.181	−0.281	−0.299	−0.204	−0.248
PM	T1	p	0.004	0.005	0.031	0.150	0.005	0.014	0.003	0.024	0.138	0.015
		Rho	0.431^**^	0.422^**^	0.333^*^	0.226	0.425^**^	0.376^*^	0.453^**^	0.347^*^	0.233	0.374^*^
	Delta^T2-T1^	p	0.028	0.035	0.306	0.457	0.406	0.035	0.095	0.289	0.372	0.376
		Rho	−0.349^*^	−0.334^*^	−0.166	−0.121	−0.135	−0.334^*^	−0.267	−0.172	−0.145	−0.144
ARoM	T1	p	0.050	0.012	0.004	0.031	0.011	0.060	0.004	0.005	0.031	0.006
		Rho	−0.304	−0.384*	−0.434^**^	−0.333^*^	−0.387*	−0.292	−0.439^**^	−0.426^**^	−0.334^*^	−0.415^**^
	Delta^T2-T1^	p	0.906	0.775	0.695	0.359	0.526	0.991	0.959	0.727	0.410	0.494
		Rho	−0.019	0.047	−0.064	−0.149	−0.103	−0.002	0.008	−0.057	−0.134	−0.111
Force	T1	p	0.007	0.001	0.000	0.019	0.001	0.009	0.000	0.000	0.021	0.000
		Rho	−0.413^**^	−0.504^**^	−0.547^**^	−0.360^*^	−0.506^**^	−0.398^**^	−0.563^**^	−0.547^**^	−0.355^*^	−0.529^**^
	Delta^T2-T1^	p	0.383	0.260	0.121	0.287	0.191	0.521	0.453	0.127	0.287	0.256
		Rho	0.142	0.183	0.249	0.173	0.211	0.104	0.122	0.245	0.173	0.184
Velocity	T1	p	0.001	0.000	0.000	0.018	0.000	0.002	0.000	0.000	0.014	0.000
		Rho	−0.488^**^	−0.563^**^	−0.515^**^	−0.362^*^	−0.540^**^	−0.459^**^	−0.600^**^	−0.516^**^	−0.375^*^	−0.524^**^
	Delta^T2-T1^	p	0.125	0.162	0.782	0.741	0.838	0.251	0.657	0.843	0.774	0.966
		Rho	0.247	0.226	0.045	0.054	0.033	0.186	0.073	0.032	0.047	0.007

Behavioral assessments: FMA: Fugl-Meyer Upper Limb Motor assessment; kUDT: kinaesthetic Up-Down test; BBT: Box and Block test; PM: position matching; ARoM: active range of motion. Rho: Spearman’s Rho. Tract templates (SMATT atlas): S1: S1 Tract; M1: M1 Tract; SMA: SMA Tract; preSMA: preSMA Tract; PMd: PMd Tract.

* Significant correlation *p* < 0.05.

** Significant correlation *p* < 0.01.

**Table 3:** Loss of SSEP: significant group differences between the group of patients with loss of SSEP vs. the group of patients with preserved SSEP (2 sample *t*-tests for normally distributed data and Mann–Whitney U tests for not normally distributed data).

	Absolute overlap			Weighted lesion load			kUDT T1	Box and Block T1
	S1	M1	SMA	S1	M1	SMA		
Mann–Whitney-U	31	33	62,5			61	41,5	65
Cohen’s d				29.7	31.8			
Z/T	Z = −3.039	Z = −2.951	Z = −1.660	T = 5.165	T = 3.710	Z = −1.730	Z = −3.127	Z = −2.021
*p*	0.0008^**^	0.0011^**^	0.0498^*^	<0.001^**^	<0.001^**^	0.0440^*^	0.0009^**^	0.0206^*^
Mean - loss of SSEP	1.51 ml ± 0.93	1.63 ml ±0.79	0.45 ml ±0.44	86.4% ±17.0	79.7% ±17.4	53.3% ±41.2	0.90 ± 1.29	8.8 ± 11.49
Mean - SSEP preserved	0.46 ml ±0.57	0.73 ml ± 1.05	0.23 ml ± 0.38	38.1% ±34.1	43.1% ±36.6	33.0% ±38.4	2.48 ± 0.85	23.6 ± 21.7

No significant group differences were seen for lesion loads of preSMA- and PMd-tract as well as for the other clinical and robotic assessments (FMA, ARoM, Force, and Velocity) at timepoint T1 and DeltaT2-T1. Behavioral assessments: FMA: Fugl-Meyer Upper Limb Motor assessment; kUDT: kinaesthetic Up-Down test; BBT: Box and Block test; PM: position matching; ARoM: active range of motion. Tract templates (SMATT atlas): S1: S1 Tract; M1: M1 Tract; SMA: SMA Tract.

* Significant correlation *p* < 0.05.

** Significant correlation *p* < 0.01.

### 2.4. Lesion analysis

#### 2.4.1. Lesion delineation and normalisation

For lesion analysis of MRI data we used the images with the strongest contrast difference between the ischemic lesion and normal tissue; in most cases these were the diffusion weighted images (DWI) for near-term imaging (up to 5 days after stroke) and FLAIR images for later acquired images. First, the individual images were manually reoriented to AC-PC plane and the reorientation parameters were applied to all other images used for normalisation. Lesion maps were semi-automatically delineated by using the Clusterize Toolbox for SPM 8 in the modified version that also supports inclusion/processing of CT images (de Haan et al., 2015); every slice of the lesion map was checked and adjusted with the supplement tools of the Clusterize Toolbox when necessary. The examiner was naïve to the clinical profiles of the patients at the time of lesion mapping. The lesion maps were normalized with Clinical Toolbox for SPM 8.^1^ The spatial position of the resulting normalized lesion map was subsequently checked for each individual by comparison with the respective normalized structural scan and the ch2better template. In case of inconsistencies, lesion maps were manually adjusted and corrected by using MRIcron Software (Rorden and Brett, 2000; https://www.nitrc.org). For left-sided lesions the maps were flipped onto the right hemisphere to increase the statistical power of identifying lesion patterns independent of the hemisphere (Kessner et al., 2019). An overlap image with all lesion maps (all left-sided lesions are flipped to the right side) was calculated with MRIcron to give an overview of the lesion localisations.

#### 2.4.2. Voxel-based lesion-symptom mapping

Voxel-based lesion-symptom mapping (VLSM) was applied to investigate structural correlates of the behavioral motor and proprioceptive impairments and of the neurophysiological data (SSEP). Statistical non-parametric analyses were computed by using non-parametric mapping software (NPM, available with MRIcron software) with Brunner-Munzel tests (10,000 permutations) for continuous behaviour of the clinical assessments (FMA, kUDT, BBT) and the robotic assessments (PM, Force, ARoM, Velocity) for results at timepoint T1 and Delta^T2-T1^. For analysis of the neurophysiological data (SSEP) we also used the Brunner-Munzel test (10,000 permutations) for binary behaviour (patients with loss of SSEP (*n* = 10) vs. patients with preserved SSEP (*n* = 23) even if the latency was delayed or the amplitude reduced). For 12 patients it was not possible to collect SSEPs.

Only significant results with a very conservative thresholding *p* < 0.05, controlled for multiple comparisons with family wise error (FWE), are reported. Statistically significant voxels were visualized on the mni152 template in MRIcroGL (See footnote 1), setting the lower bound intensity value of each resulting map to the specific critical z-value of the FWE-analysis.

#### 2.4.3. Statistical region of interest analysis

Additional to the whole brain analysis, we performed different statistical ROI-based analyses. The specialised software NiiStat^3^ in MATLAB (MathWorks, Natick, MA) allows for non-parametric mapping of explicit ROIs with behavioural data. Previous studies found evidence for a correlation of sensory deficits with lesion of the CST (Findlater et al., 2019; Ingemanson et al., 2019a) as well as with cortical areas, especially with the primary sensory cortex, the supramarginal gyrus, insula and the superior temporal cortex (Kenzie et al., 2016; Kessner et al., 2019). We performed analyses for the CST using the CST template of the CAT atlas (Catani and Thiebaut de Schotten, 2008; http://www.natbrainlab.com). This analysis detects the count of significant lesioned voxels in the ROI corresponding to the continuous behavioural data. This CST template is a probabilistic mask and we included in this analysis only subjects with lesions overlapping with voxels of the CST with at least 50% probability. To investigate changes in different cortical areas, we also performed an atlas-based analysis with NiiStat. This ROI-based approach uses predefined segmentations of the cortex, testing voxelwise each ROI with regard to the lesion maps and the corresponding behavioural data and reporting threshold-surviving regions. For the cortical areas we used the AICHA atlas (384 grey-matter ROIs; Joliot et al., 2015). All analyses were performed with 10,000 permutations, all reported *p*-values were thresholded <0.05, FEW corrected for multiple comparisons and one-tailed based on the *a-priori* hypothesis that brain injury leads to impaired behavioral performance. All significant results are visualized on the mni152 template in MRIcroGL.

#### 2.4.4. Lesion metrics

Stroke-related injury of CST was quantified by measuring the extent to which the patient’s lesion overlapped with corticospinal templates. To improve spatial resolution within the corticospinal tract, we used the Sensorimotor Area Tract Templates (SMATT; downloaded from http://lrnlab.org/; Archer et al., 2018). This set contains templates in standard space based on segmented cortical regions from where probabilistic tractography analyses had been conducted in 100 subjects using the highest resolution data available. We used the 5 tracts from the primary somatosensory cortex (S1), the primary motor cortex (M1), supplementary motor area (SMA), pre-supplementary motor area (preSMA) and the dorsal premotor cortex (PMd). The templates were co-registered with the NMI space. Injury to each of these 5 tracts in each subject was quantified by two different lesion metrics – the absolute overlap and a weighted lesion load (wLL) – even if a significant difference between both approaches could not be demonstrated before (Findlater et al., 2019). The overlap of each tract was equivalent to the absolute number of overlapping voxels of the tract with the individual lesion map. The wLL was calculated to account for the narrowing of the tracts in the region of the internal capsule as well as for the fact that a complete injury of a tract in the axial plane would cause complete paralysis although the absolute number of overlapping voxels could be small. To this end, in each axial z-slice the absolute number of voxels of the tract was determined as well as the number of overlapping voxels with the lesion map within this z-slice. In this way the percentage of tract injury for each z-slice could be calculated; the 10 z-slices with the highest percentile ranks (according to at least 10 mm in z direction of the tract) were averaged, thereby obtaining the wLL of the specific tract of each subject. These single values were correlated with the clinical and robotic measures. For 3 subjects, this analysis was not possible because their lesions were located below the caudal end of the tract templates (MNI z-slice<−35).

## 3. Results

### 3.1. Behavioral data

Forty-one patients completed all clinical and forty-three the robotic assessments at timepoint T2. We saw significant improvements in six assessments: FMA, kUDT, BBT, PM, ARoM, Force. No significant improvement was observed in Velocity.

### 3.2. Lesion overlap

**Figure 1:**
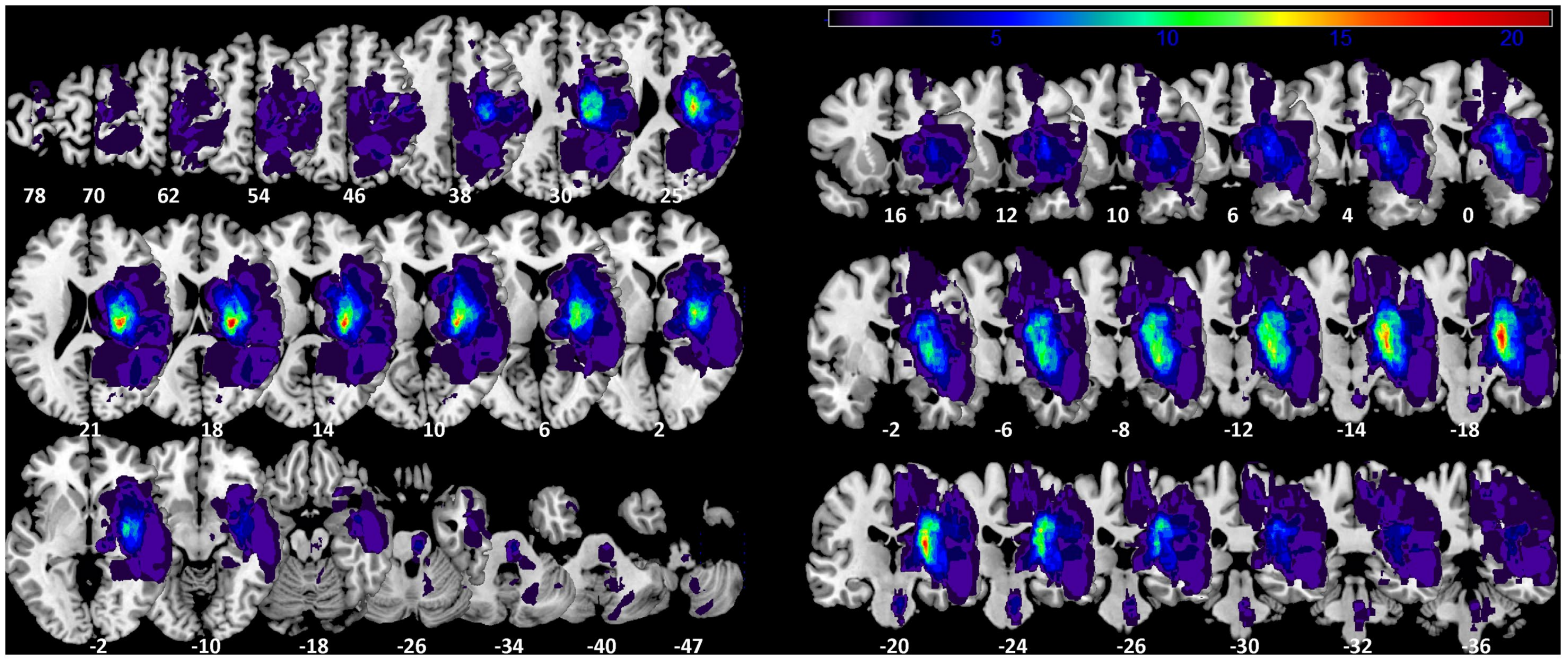
Lesion probability map. Axial and coronal slices of lesion overlap image of the 45 summed up lesion maps. Slices are labelled with the corresponding z- and y-coordinates of MNI space [left-sided lesions were flipped on sagittal plane to the right side]. The maximum overlap (21 subjects) is located at MNI coordinate 26-18 18.

We overlaid the 45 normalized lesion maps of the single subjects to better describe the maximum of lesions at the group level. This group lesion overlap map included 379,143 voxels with at least one subject’s lesion, corresponding to 379.1 ml (see Figure 1). The mean lesion volume per subject was 17.65 ml ±30.00, min: 0.50 ml; max 133.64 ml. The maximum overlap was observed in 5 voxels (lesions of 21 subjects) located in the internal capsule (CAT atlas) at MNI coordinate x = 26, y = −18, z = 18.

### 3.3. Voxel-based lesion-symptom mapping

**Figure 2:**
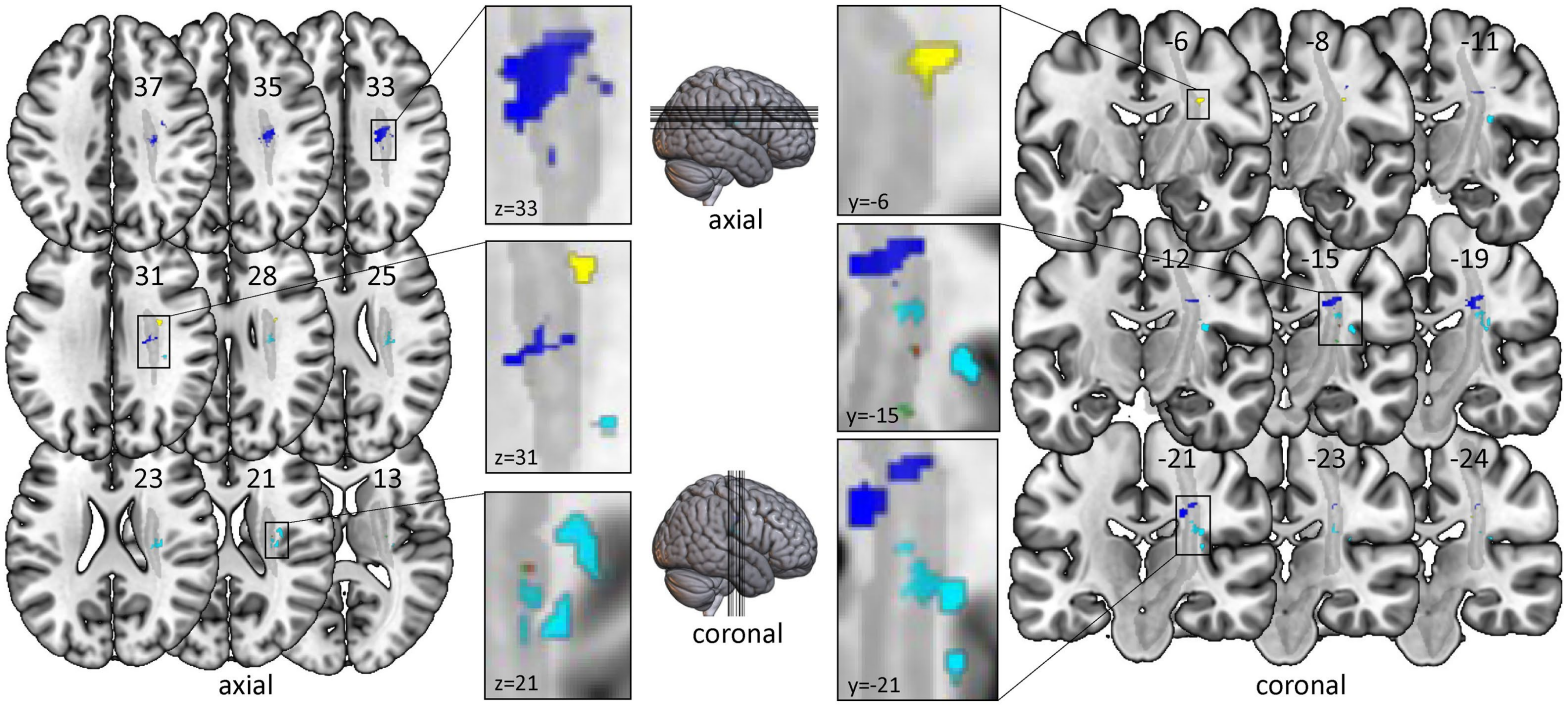
Voxel-based lesion-symptom mapping of clinical and robotic assessments of timepoint T1. All test results were analysed with nonparametric VLSM software NPM with correction for multiple comparisons using Brunner-Munzel tests with the conservative threshold of permutation family-wise error rate of 0.05. Only significant voxels with z-values over the specific z-threshold are shown. Left-sided lesions were flipped to the right side. All significant voxels are located within or very near the corticospinal tract. Color-coding for clinical assessments: Red for Fugl-Meyer Upper Limb Motor Assessment; Cyan for kinaesthetic Up-Down Test; Green for Box and Block Test. Color-coding for robotic assessments: Yellow for Position Matching; Purple for Force; Blue for Velocity. Grey: template of corticospinal tract (CAT Atlas, Natbrainlab). Results are visualized on the mni152 template in MRIcroGL.

In the VLSM analysis, we found significant relationships for all clinical and robotic assessments but ARoM at timepoint T1 (4,000 Permutations, Brunner-Munzel test, FEW corrected 0.05): FMA: threshold z > 3.891; kUDT: threshold z > 3.719; BBT: threshold z > 3.738; Position matching: threshold z > 3.891; Force: threshold z > 3.891; Velocity: threshold z > 3.891. Most of the significant voxels were located in the white matter, mainly within the CST template (see Figure 2). Only the mapping for kinaesthetic Up-Down Test showed significant voxels in cortical areas, especially in the posterior part of insula and the rolandic operculum.

For Delta^T2-T1^ - indicating the change of proprioceptive and motor impairments between timepoint T1 and T2 - we found no significant results.

### 3.4. Statistical region of interest analysis

**Figure 3:**
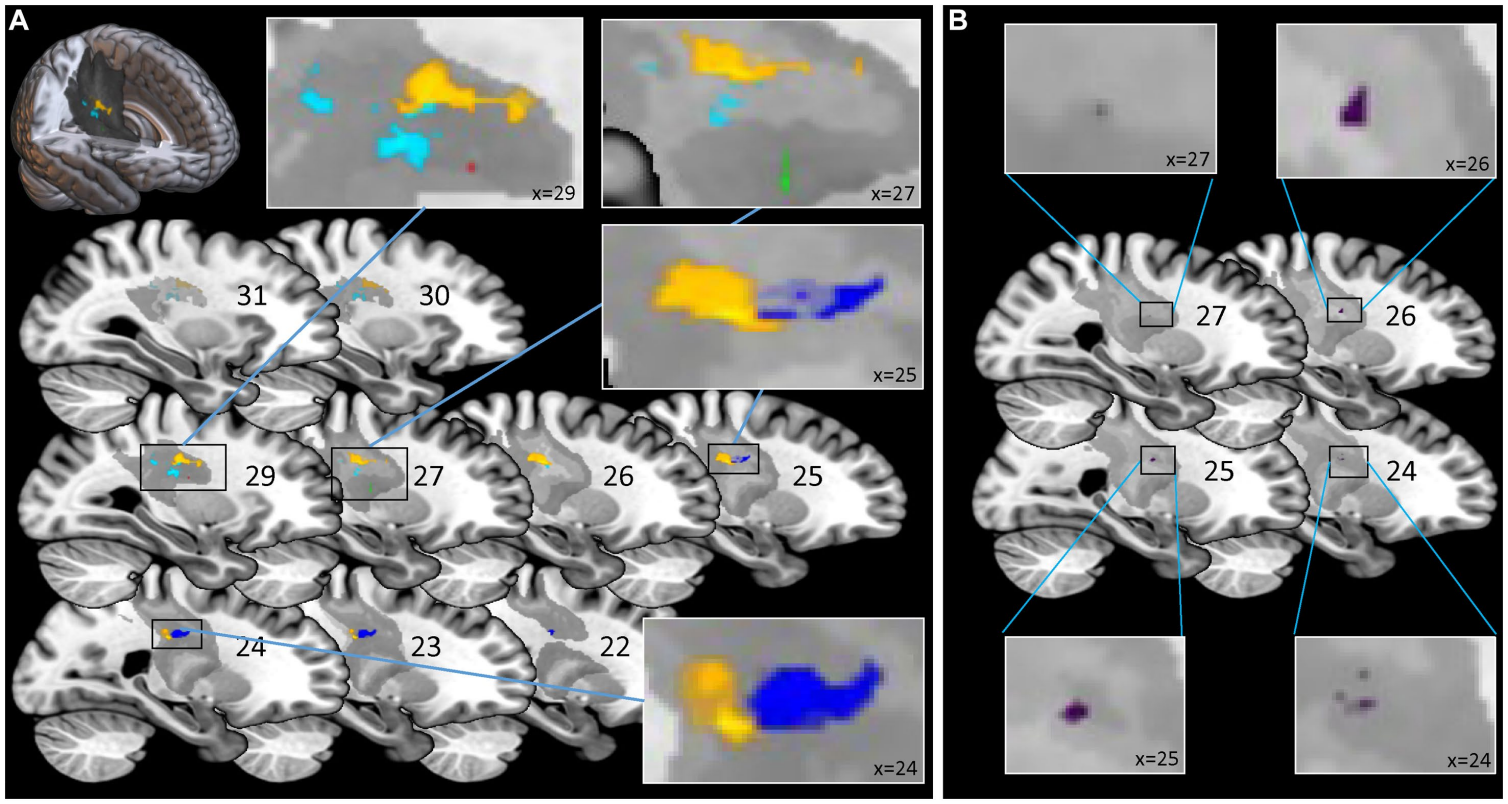
Statistical Region of Interest (ROI) Analysis for corticospinal tract: voxel-based lesion symptom mapping of clinical and robotic assessments for the explicit ROI of corticospinal tract (CAT Atlas, Natbrainlab). **(A)** Analysis for timepoint T1: significant results for Fugl-Meyer Upper Limb Motor Assessment, kinaesthetic Up-Down Test, Box and Block Test, Position Matching and Velocity. Significant voxels associated with assessments specific for proprioception (kinaesthetic Up-Down Test; Position Matching) were located more posterior than voxels corresponding to assessments associated with motor tasks. **(B)** Analysis for Delta^T2-T1^ - indicating the change of impairments between timepoint T1 and T2 – only showed significant results for the Box and Block Test located in the anterior part of CST. Color-coding for assessments **(A)** Red for Fugl-Meyer Upper Limb Motor Assessment at T1; Cyan for kinaesthetic Up-Down Test at T1; Green for Box and Block Test at T1; Yellow for Position Matching at T1; Blue for Velocity at T1; **(B)** Purple for Box and Block Test Delta^T2-T1^.

The findings of the ROI analysis are in line with the results of the NPM analysis. Testing for the region of interest CST we found significant results for all clinical and robotic assessments but ARoM and Force at timepoint T1 (see Figure 3A): kUDT: 151 voxels surviving threshold z > 3.946; FMA: 1 voxel surviving threshold z > 3.433; BBT: 13 voxels surviving threshold z > 3.230; Position matching PM: 345 voxels surviving threshold z > 3.544; Velocity: 94 voxels surviving threshold z > 2.949 (voxelsize 1 × 1 × 1 mm). The significant voxels of the assessments specific for proprioception were located in the posterior part of CST. For Delta^T2-T1^ - indicating the change of proprioceptive and motor impairments between timepoint T1 and T2 - we found significant results only for BBT [17 voxels surviving threshold z > 3.123 in the anterior part of CST template (see Figure 3B)].

For the atlas-based analysis of cortical areas (AICHA templates) only the kUDT for timepoint T1 showed significant results; various regions associated with processing of sensory stimuli survived z-threshold: Postcentral cortex-1, z = 3.428; Postcentral cortex-3, z = 3.053; Rolando sulcus-1, z = 2.952; Supramarginal gyrus-1, z = 3.192; Insula posterior-1, z = 3.805; Rolandic operculum-2, z = 3.241. All other clinical and robotic assessments showed no significant results for timepoint T1 or Delta^T2-T1^.

### 3.5. Lesion metrics

Both used lesion metrics - the absolute overlap and weighted lesion load – yielded identical results concerning significant nonparametric correlations of lesion metrics and behavioral assessments (see Table 2). For timepoint T1 most behavioural assessments were correlated with most tract templates. For Delta^T2-T1^, however, only the correlation of change of Position Matching (PM) over time with the lesion metrics of tract S1 and M1 showed significant results, suggesting that the lesion of these tracts may be a negative predictor of recovery of kinaesthetic perception (see Table 2). Since the S1 tract and the M1 tract of the SMATT atlas overlap to a large extent from corona radiata down to the midbrain, the correlation unfortunately cannot be assigned to the S1 or M1 tract. On the basis of this correlation it is not possible to decide whether the result is due to a lesion of ascending axons to S1 or descending axons from M1 or both. It should be noted, however, that the more anterior located tracts such as SMA tract, preSMA tract and PMd tract did not show any significant correlations with Delta^T2-T1^ for PM. It can therefore be assumed that lesions next to posterior parts of corticospinal connections are associated with poor recovery of kinaesthetic perception.

### 3.6. Loss of SSEP

Patients with loss of SSEP showed lower scores in kUDT and BBT at timepoint T1 than patients with preserved SSEP (see Table 3). There was a trend for group difference concerning the Delta^T2-T1^ of Position matching (*p* = 0.064, T = −1.564; Delta^T2-T1^ of mean error for the group loss of SSEP: 0.32° ± 3.73°; Delta^T2-T1^ of mean error for the group SSEP preserved: 2.70° ± 4.13°). The other clinical and robotic assessments (FMA, ARoM, Force, Velocity) at timepoint T1 and for Delta^T2-T1^ were not different for both groups.

**Figure 4:**
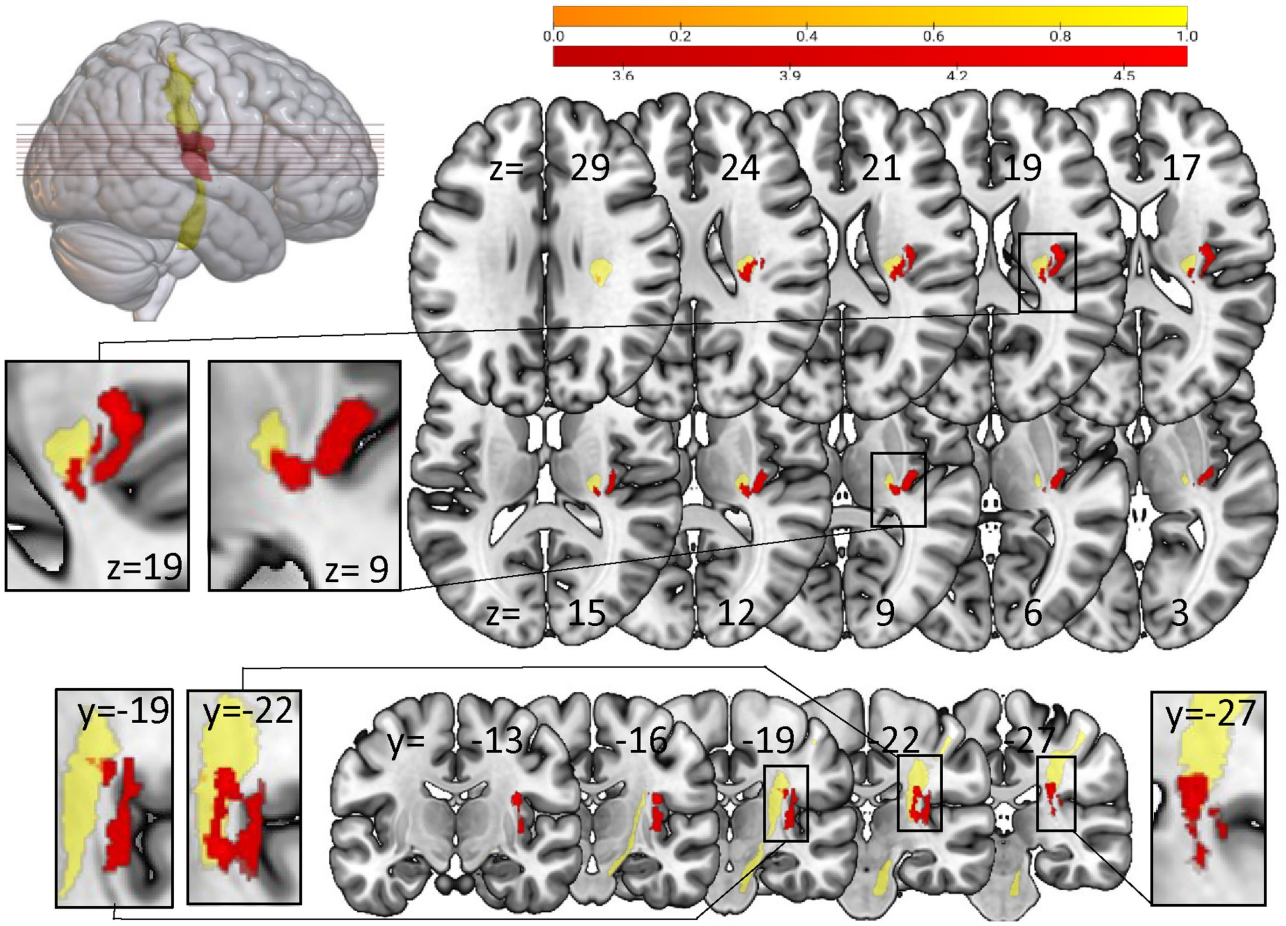
Voxel-based lesion-symptom mapping analysis with NPM concerning loss of SSEP vs preserved SSEP. Significant voxels (red) associated with the loss of SSEP (Brunner-Munzel test, permutation FWE corrected for *p* < 0.05). Yellow: S1 tract template of SMATT atlas. Left-sided lesions were flipped to the right side. Results are visualized on the mni152 template in MRIcroGL. Slices are labelled with the corresponding x-, y-, and z-coordinates of MNI space.

The VLSM analysis in patients with loss of SSEP vs. patients with preserved SSEP showed significant voxels in cortical areas, especially in the posterior part of insula and the rolandic operculum and in the area of the corticospinal tract (10,000 Permutations, Brunner-Munzel test, threshold z > 3.4751, FEW corrected 0.05; z-value up to 4.804), overlapping clearly with S1 tract (see Figure 4, S1 tract template of SMATT atlas).

Additionally the group of patients with loss of SSEP showed significantly higher lesion loads in S1-, M1- and SMA-tracts (absolute overlap and weighted lesion load). No statistically significant group differences were seen for lesion loads of the preSMA- and PMd-tract.

## 4. Discussion

The objective of the study was to investigate the relationship between lesion location and the associated motor and proprioceptive impairments in subacute stroke patients - and further on to look for potential predictors of proprioceptive recovery. Proprioceptive deficits affect not only sensory functions but are also relevant for motor recovery after stroke (Kessner et al., 2019; Zandvliet et al., 2020). Identifying lesion locations associated with poor proprioceptive recovery could help to better tailor rehabilitation programs.

In our study, we measured proprioception in the area of the finger using clinical and robotic assessments. It could be argued that these assessments are not conclusive in terms of proprioceptive impairments in other areas of the body. However, Tyson et al. (2008) found no differences in proprioceptive deficits between arm and leg or between proximal and distal joints in a large cohort of stroke patients. We can therefore assume that the measurement of proprioception in the finger area hints towards more general proprioceptive deficits.

For the motor outcome after stroke it is well known that lesions in the corticospinal tract are closely associated with poor motor recovery (Lin et al., 2019; Liu et al., 2020). However, if at all, there is only scarce evidence that a specific lesion or the lesion load in the CST may also predict poor proprioceptive recovery (Findlater et al., 2019). The location of the lesions in the current patient sample with subacute stroke was similar to other studies (Kessner et al., 2016; Meyer et al., 2016) and we did observe a significant improvement after rehabilitation in six of the sensory and motor assessments (only Velocity did not increase). Furthermore, the present results show that motor and sensorimotor impairments were associated with mainly white matter lesions in the area of the CST. The specific lesions associated with proprioceptive deficits were located dorsal to the posterior internal capsule joined to the posterior part of the CST. This suggests that such lesions might also predict poorer recovery of proprioceptive functions.

### 4.1. White matter lesions

In the VLSM analysis we found significant results for six assessments in voxels located in the white matter, in or near the CST template. Two of the assessments (kUDT and PM) illustrate exclusively proprioceptive deficits, two others measure predominantly motor parameters (Force and Velocity). The last two quantify motor deficits but they are also affected by proprioceptive deficits (FMA; BBT; Ingemanson et al., 2019b). While the allocation of lesions to motor deficits are well-studied (Lo et al., 2010; Olafson et al., 2021), there is only little and quite heterogeneous data on the association between the precise lesion location and somatosensory deficits after stroke. Two studies report mainly cortical regions such as the primary and secondary somatosensory cortex (S1, S2) and the dorsal insular cortex being associated with somatosensory deficits (Kenzie et al., 2016; Kessner et al., 2019), while other studies mention only white matter regions (Carter et al., 2012; Findlater et al., 2019) or both white matter and cortical lesions (Meyer et al., 2016; Ingemanson et al., 2019a).

For the robotic assessment of proprioceptive deficits (PM) the VLSM analysis revealed significant voxels restricted to the white matter. Only for the clinical kinaesthetic Up-Down test we found – in addition to white matter lesions – associated regions in the posterior part of insula und the rolandic operculum. These regions are well established in the processing of somatosensory input (Mazzola et al., 2012). Interestingly, these cortical regions were only associated with kUDT – a test with a coarse nominal scale – and not with PM, which is described as more sensitive to proprioceptive deficits (Ingemanson et al., 2019a).

Nevertheless, the fact that our analyses identified almost exclusively white matter lesions does not rule out cortical lesions as a cause for motor and proprioceptive deficits. The findings rather point to the fact that the maximal lesion overlap – like in other stroke patient samples (Kessner et al., 2016; Meyer et al., 2016) – is located in the internal capsule. Taken together, it appears that the most common lesions in stroke patients with motor and proprioceptive deficits are located in the white matter.

### 4.2. ROI-analysis for CST

Lesions in the CST were shown to be associated with motor (Zhu et al., 2010; Liu et al., 2020) as well as with somatosensory (Carter et al., 2012; Meyer et al., 2016) impairments. Our ROI analysis of the CST identified significant voxels in the CST template for all clinical assessments as well as for two robotic parameters (PM and Velocity) at timepoint T1. The related regions showed nearly no overlap (see Figure 3).

Consistent with the expectation, the regions correlated to the pure proprioceptive deficits (kUDT and PM) were located in more posterior parts of the CST template than those associated with proper motor deficits. The corticospinal tract includes fibers descending from the motor areas as well as from the somatosensory cortex S1/S2 (Liu et al., 2018). The posterior part of the internal capsule comprises descending fibers of the corticospinal tract (Qian and Tan, 2017) but also ascending fibers from thalamocortical projections to the somatosensory cortex (Hong et al., 2010). This reflects that it is difficult to disentangle fibers carrying motor from the proprioceptive information in the course of the CST.

The pre-existing CST template used in our study is derived from DTI and thus includes ascending and descending fibers. It can be assumed that the posterior part of this template in particular, which also penetrates the posterior part of the internal capsule, contains ascending fibers.

Proprioceptive deficits are expected to be associated with lesions of the ascending fibers and hence with posterior areas of the CST template. Therefore, the identified lesion location correlated with proprioceptive deficits observed in the current study is well in line with the general neuroanatomical knowledge.

To better capture the differences between the different parts of the CST template, we used the subfractions associated with the primary sensory cortex S1 and the motor areas M1, SMA, pre-SMA and PMd (SMATT templates; Archer et al., 2018) for further lesion load analysis. A clear separation between ascending and descending fibers was not possible either, since S1 and M1 tracts overlap in their course. However, the S1 tract is clearly located in the posterior part of the CST template and does not overlap with SMA, pre-SMA and PMd tracts located more anterior. Therefore, it can be assumed that lesions in the area of the S1 tract affect ascending fibers.

### 4.3. Loss of SSEP

In our study patients with loss of SSEP performed worse in kUDT and BBT. This is in line with the literature. Typically, loss of SSEP is not only correlated to somatosensory deficits, but patients with loss of SSEP also show significantly poorer results in motor assessments (Yoon et al., 2021). In the VLSM analysis we identified areas of lesion associated with loss of SSEP. These were located in the posterior part of the CST template overlapping mainly with the S1 tract. This matches well with the ROI analysis for the CST template in which patients with proprioceptive deficits (kUDT and PM) exhibited lesions in the same area. The loss of SSEP was also associated with lesions in secondary somatosensory areas as the rolandic operculum and the posterior part of insula. These areas overlapped also with the region associated with poor results for kUDT in the whole brain VLSM analysis. Taken together, lesions in the posterior part of the CST template and secondary somatosensory areas induce loss of SSEP and proprioceptive deficits.

Furthermore, we observed that patients with loss of SSEP showed a trend for less recovery of proprioceptive function assessed by the robotic test PM. This may suggest that lesions in the posterior part of the CST template could predict poorer outcome of proprioceptive recovery. This is relevant for activities of daily life since somatosensory impairments impede regaining motor capacities in patients with hemiplegia (Yoon et al., 2021) and it sums up to evidence that the assessment of SSEP may predict ADL recovery (Tzvetanov and Rousseff, 2003; Lee et al., 2015; Hwang et al., 2016).

### 4.4. Predictor of recovery

All assessments but Velocity showed a significant improvement for Delta from timepoint T1 to T2, indicating recovery to some extent. The whole brain VLSM analysis did not uncover any associations with the recovery. However, the ROI analysis for CST revealed a region in the anterior part of the CST associated with improvement in Box and Block test. The BBT mainly reflects motor and less proprioceptive functions. Importantly, lesions associated with proprioceptive deficits were rather observed in the posterior part of the CST template.

The further analysis of lesion metrics revealed that rehabilitation-related changes for the robotic proprioceptive parameter PM showed a significant modulation for subparts of the corticospinal tract. This is in line with the study of Findlater (Findlater et al., 2019) who also found a correlation of the proprioception measure related to clinical changes and lesion metrics in the CST. By analysing the lesion loads of the subparts of CST we could further specify the localisation: only the lesion loads of S1 and M1 tracts correlated with poor proprioceptive recovery. The lesion load for the other three motor tracts of SMA, pre-SMA und PMd were unrelated. The S1 and M1 tracts widely overlap and are clearly located in the posterior part of the CST. It appears that lesions in the posterior part of the CST could predict a poor recovery of proprioception.

Furthermore, the loss of SSEP was also associated with lesions in the posterior part of the CST template overlapping with S1 tract and, at the same time, patients with loss of SSEP showed a trend for a poor recovery concerning PM. Thus, the current study provides hints that the specific lesion in the posterior part may predict worsened recovery of proprioception.

### 4.5. Limitations

The results of the current study are mostly well in line with the literature and provide some novel aspects. Nevertheless, they need to be interpreted with care. The segregation of motor and proprioceptive functions in the CST structural imaging data is difficult mainly because of the underlying resolution of the voxels. Differences in individual anatomy at small scale cannot be detected. Higher resolutions that can be achieved with higher magnetic field strength might provide a solution to this problem. At present, we cannot completely rule out that other white matter tracts might have contributed to the presented result pattern.

## Data availability statement

The raw data supporting the conclusions of this article will be made available by the authors after complete anonymization.

## Ethics statement

The studies involving humans were approved by Ethics Commission of Baden-Württemberg (F-2016-126), Landesärztekammer Baden-Württemberg, Ethik-Kommission, Liebknechtstraße 33, 70565 Stuttgart. The studies were conducted in accordance with the local legislation and institutional requirements. The participants provided their written informed consent to participate in this study.

## Author contributions

TH, MZ-M, RG, OL, JL, and MS conceptualized and designed the research. TH, MZ-M, and CS were responsible for data collection. TH and MS undertook the statistical analysis and wrote the first draft of the manuscript. TH, MZ-M, JL, and MS interpreted the results and edited the final manuscript. JL and MS contributed equally to this work and share last authorship. All authors contributed to the article and approved the submitted version.

## Footnotes

^1^ https://www.nitrc.org

^3^ https://www.nitrc.org/projects/niistat
